# Primary Retroperitoneal Ganglioneuroma: A Retrospective Cohort Study of 32 Patients

**DOI:** 10.3389/fsurg.2021.642451

**Published:** 2021-05-21

**Authors:** Jianchun Xiao, Zixuan Zhao, Binglu Li, Taiping Zhang

**Affiliations:** ^1^Department of General Surgery, Peking Union Medical College Hospital, Chinese Academy of Medical Sciences, Beijing, China; ^2^School of Medicine, Tsinghua University, Beijing, China

**Keywords:** retroperitoneal ganglioneuroma, diagnosis, surgery, surveillance, outcome

## Abstract

**Purpose:** To investigate the clinical characteristics, diagnosis, differential diagnosis, therapy options, and outcomes of retroperitoneal ganglioneuroma.

**Methods:** In this retrospective study, we collected and analyzed the clinical data of 32 patients diagnosed with retroperitoneal ganglioneuroma and admitted to Peking Union Medical College Hospital from October 2012 to August 2019.

**Results:** Among our 32 cases with retroperitoneal ganglioneuroma, the male-to-female ratio was 1:3 and the mean age was 35. Only 25% of the cases presented with abdominal pain while more than 65% had no specific symptoms. The masses could be found through physical examination in only five patients. Most of the tumors are located near the renal area. They were usually single and displayed an embedded growth pattern with diameters <10 cm, clear borders, and soft texture. For radiological imaging, the majority of tumors demonstrated soft tissue density with mild-to-moderate enhancement on CT imaging and showed hypoecho with moderate blood flow signals in ultrasound. No significantly abnormal laboratory examinations were found in most patients. Of all the 32 patients, 2 chose surveillance after biopsy due to difficulties in operation, while others chose surgical resection. The mean follow-up time was 15.8 months among 26 patients. The tumor remained stable in the surveillance cases. Residual tumors were found in four cases receiving operations with no progress and discomfort. No recurrence was seen in all patients.

**Conclusions:** The retroperitoneal ganglioneuroma is a benign tumor without specific clinical manifestations or significant laboratory findings. Typically, it is shown as low density with a clear border and an embedded growth pattern in radiological imaging. The overall prognosis is good. Surgery is an effective approach with possible severe complications. Incomplete resection or surveillance can be considered for some cases where complete resection is difficult to achieve.

## Introduction

Ganglioneuroma (GN) is a rare benign tumor of neural crest origin arising from the sympathetic nervous system. It is a mature and well-differentiated subtype of peripheral neuroblastic tumors (pNTs) that are classified into four categories: neuroblastoma (NB), ganglioneuroblastoma-nodular (GNB-N), ganglioneuroblastoma-intermixed (GNB-I), and GN in International Neuroblastoma Pathology Classification (INPC) (1). Histologically, GNs are mainly composed of mature Schwannian stroma and ganglion cells, and they are commonly diagnosed at an older age compared with other subtypes of pNTs (2).

The majority of GNs arise as primary lesions, but secondary GNs can be found differentiated from NB spontaneously or induced by treatment (3). Theoretically, GNs can be found anywhere alongside the sympathetic system. Common sites include posterior mediastinum (41.5%), retroperitoneum (37.5%), and adrenal gland (21%) (4). GNs in other areas such as cervical region, trigeminal nerve, colon, and skin are very rare (5–8). Retroperitoneal GN only constitutes 0.72 to 1.6% of all the primary retroperitoneal tumors (9). The patients are usually asymptomatic and have excellent prognosis, while sometimes the large mass may compress neighboring structures, causing corresponding symptoms and even damage (10). For lack of symptoms, clinical diagnosis mainly depends on radiological findings, which can be misleading sometimes and require confirmation by histopathological examination and immunohistochemical staining. The gold standard for GN diagnosis is still pathology.

Previous reports of retroperitoneal GN are mainly single cases and only a few studies summarized some characteristics of the masses. As their clinical behaviors and features are still poorly understood, we retrospectively analyzed the clinical data of 32 patients with retroperitoneal GNs including several hard cases and summarized their features, diagnosis, treatment strategies, and outcomes to provide more experience for diagnosis and therapy of retroperitoneal GNs.

## Materials and Methods

In this retrospective study, we collected and analyzed the medical records of 32 cases diagnosed with retroperitoneal GN in Peking Union Medical College Hospital (PUMCH) from October 2012 to August 2019. Patient data included sex, age, manifestations, physical examination, laboratory and radiological findings, treatments, outcomes, and follow-up. Laboratory examination mainly included endocrine tests (examinations on renin–angiotensin–aldosterone system, sympathetic–catecholamine system, and sex hormones), tumor antigens such as CEA, CA125, CA19-9, CA724, NSE, and AFP, as well as metabolic indexes. Radiological findings included the location, size, number, shape, border, density, neighboring structures, the blood flow information, as well as enhancement performances. For pathological reports, the sizes of tumors, gross macroscopic findings, immunohistochemical staining, and pathological diagnosis were collected. Clinical diagnoses of retroperitoneal GN were mostly based on radiological findings, and the definite diagnoses relied on histopathological examinations.

For surgical procedure of laparotomy, incision position was dependent on the tumor location, mostly through the rectus abdominis. Firstly, the surgeons cut into the abdominal cavity layer by layer and explored the tumor. Then, they opened the peritoneum, protected and separated the neighboring structures, and exposed the mass. Next, fibrous connective tissue involved by the mass was removed and the tumor was freed along the tumor capsule until it was resected. Last, the surgeon stopped the bleeding, placed a drainage tube on the wound, led it out of the abdomen with another opening, and closed the abdomen layer by layer. For laparoscopic operation, normally three ports were placed. Similarly, the tumor was exposed from the adjacent organs and vessels, and then it was freed along the capsule and finally removed. Besides, one of the laparoscopic cases was achieved through robotic technique.

This study did not directly involve patient subjects and is exempt from ethical committee approval. Informed consent was waived considering it is a retrospective study. Descriptive data were summarized by frequencies and percentages.

## Results

### Clinical Data

This study collected the clinical data of 32 patients with retroperitoneal GN admitted to PUMCH from October 2012 to August 2019 (Table 1). The sex ratio was 1:3 with 8 males and 24 females, and the mean age was 35 years old (range: 15–62). Of all the 32 patients, most patients (65.625%) had no specific symptoms. Twenty-five percent of the patients presented with abdominal pain, and two patients exhibited weight loss of more than 2.5 kg. One patient suffered from other tumor-mass effects, which presented as hand numbness and neck pain resulting from the compression of nerves by the mass. No patient had fever or fatigue, and the weight loss was <5 kg/month. Three patients had a history of hypertension, and 25% of the patients had metabolic diseases including diabetes, hyperlipemia, and hyperuricemia. As for previous surgery history, 31.25% of the patients went through abdominal or pelvic surgeries including appendectomy and gynecologic surgery. Besides, among the male ones, 7 patients (87.5%) had smoking and drinking history. One patient had long-time connective tissue disease (CTD) including SLE and Sjogren syndrome. By physical examination, abdominal masses varied in texture and mobility were only found in five patients with no haphalgesia. The retroperitoneal GNs were mostly single (87.5%), and two GNs were found in 12.5% of the cases, so the 32 cases actually possessed 36 tumors. As for treatment, except for two patients who underwent biopsy and chose regular reexamination and surveillance, the other 30 patients received tumor resection.

**Table 1:** Clinical data of 32 retroperitoneal ganglioneuroma cases.

**Clinical characteristics**	**Number (percentage)**
**Sex**	
Male	8 (25%)
Female	24 (75%)
Mean age (years)	35 (range: 15–62)
**Manifestations**	
Abdominal pain	8 (25%)
Weight loss >2.5 kg in a month	2 (6.25%)
Hand numbness and neck pain	1 (3.125%)
No specific symptoms	21 (65.625%)
**Past history**	
Hypertension	3 (9.375%)
Hyperlipemia	5 (15.625%)
Diabetes	2 (6.25%)
Appendectomy	3 (9.375%)
Cesarean section	3 (9.375%)
Other abdominal/pelvic surgery	4 (12.5%)
CTD	1 (3.125%)
**Physical examination**	
Tumor mass	5 (15.625%)
No finding	27 (84.375%)
**Tumor number**	
1	28 (87.5%)
2	4 (12.5%)
**Treatment**	
Surgery	30 (93.75%)
No	2 (6.25%)

### Tumor Features

Based on tumor gross observations and radiological findings, these 36 retroperitoneal GNs exhibited some characteristic features (Table 2). Most of the tumors displayed an embedded growth pattern, which can compress neighboring organs. These abdominal masses were varied in diameter, ranging from 2.8 cm to 15.6 cm. The precise sizes of masses were measured by ultrasound, radiography, or gross findings, which are shown in Table 2. As we can see, more than 85% of the tumors were <10 cm. Forty-two percent of the tumors were regular in shape and around 92% displayed a clear border. Macroscopically, around 2/3 of the tumors were soft, while 22.2% were tough and 13.9% were hard in texture. Their sections were majorly gray pink and fine.

**Table 2:** Features of 36 retroperitoneal GN tumors.

**Tumor features**	**Number (percentage)**
**Tumor size**	
<5 cm	14 (38.89%)
5–10 cm	17 (47.22%)
≥10 cm	5 (13.89%)
**Tumor shape**	
Regular	15 (41.67%)
Irregular	21 (58.33%)
**Tumor border**	
Clear	33 (91.67%)
Not clear	3 (8.33%)
**Tumor texture**	
Tough	23 (63.89%)
Soft	8 (22.22%)
Hard	5 (13.89%)

### Radiological Findings

So far, screening and clinical diagnosis of GN mainly depends on radiography. With the development of imaging methods and techniques, it would be easier for us to distinguish benign from malignant tumors as well as identify the possible origin site. However, as the tumors can exhibit untypical features, sometimes the radiological examination can be inaccurate and even misleading. The radiological methods and the findings are shown in Table 3. As we can see, to identify the tumor type, there were roughly six imaging methods: computed tomography (CT) imaging (*n* = 30), ultrasound (*n* = 22), magnetic resonance imaging (MRI, *n* = 10), PET/CT (*n* = 3), somatostatin receptor (SSR) imaging (*n* = 14), and MIBG adrenal medulla imaging (*n* = 7).

**Table 3:** Radiological findings of 32 retroperitoneal GN patients.

**Tumor features**	**Number (percentage)**
**CT**	30
**Density (plain CT)**	
CT values	31.78 (21–45, *n* = 16)
Uniform	23 (76.67%)
Uneven	6 (20%)
Cystic component	8 (26.67%)
Calcification	4 (13.33%)
Not found	1 (3.33%)
**Enhancement**	
No	8 (29.63%)
Enhanced	19 (70.37%)
**Diagnosis**	
Consider neurogenic tumor	14 (66.67%)
Malignancy	6 (28.57%)
**Other tumors**	
Adrenal	3
Lymphatic	4
Embryonal	1
Leiomyosarcoma	1
Liposarcoma	3
Adnexa	1
**Ultrasound**	22
Hypoecho	21 (95.45%)
Echoless	1 (4.55%)
Hyperechogenic components	5 (22.72%)
**Blood flow signals**	
Yes	10 (50%)
No	10 (50%)
**MRI**	10
Normal plain MRI	1 (10%)
**T1**	
Hypointensity	4 (66.67%)
Isointensity	2 (33.33%)
**T2**	
Isointensity	2 (33.33%)
Hyperintensity	4 (66.67%)
**DWI**	
Hyperintensity	4(100%)
Enhanced	6(100%)
**Somatostatin receptor imaging**	14
No expression	13 (92.86%)
Mildly expressed in the margins	1 (7.14%)
**MIBG adrenal medulla imaging**	7
Normal	7 (100%)
**PET/CT**	3
SUV	1.4–2.2
Moderately elevated	1 (33.33%)

**Figure 1:**
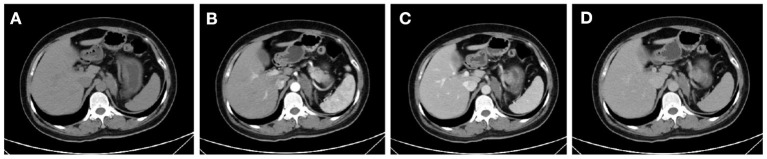
CT showed an irregular mass of soft-tissue density above the left kidney in the plain scan **(A)**. In enhanced scan, the arterial phase **(B)**, venous phase **(C)**, and delayed phase **(D)** showed uneven and mild enhancement.

**Figure 2:**
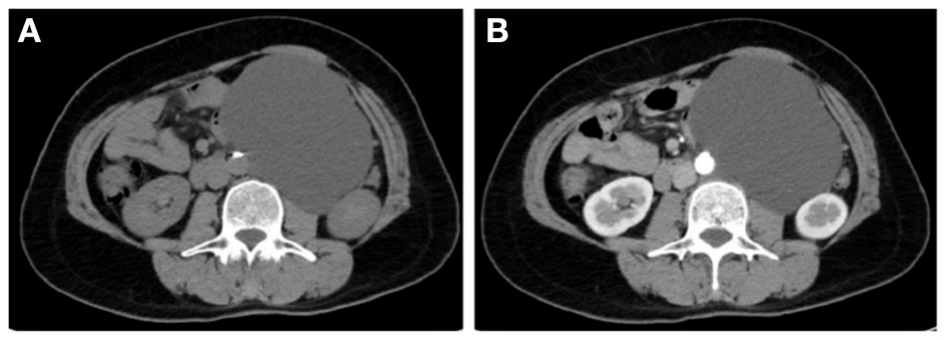
CT showed a large thin-walled cystic mass of low density **(A)** with no enhancement **(B)**.

**Figure 3:**
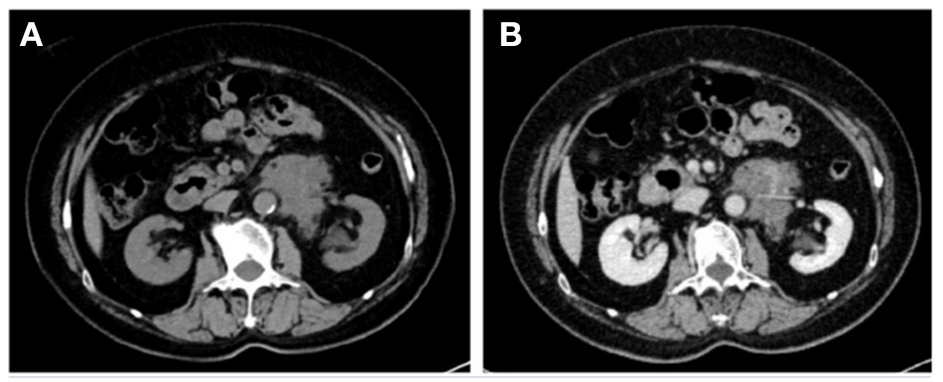
CT showed an irregular retroperitoneal mass of mixed density with coarse margin between the abdominal aorta and the left renal hilum in plain CT **(A)**. The mass was shown as heterogeneous enhancement in the delayed phase **(B)**.

Among them, CT imaging was the most common method, followed by ultrasound. In plain CT, the density of GNs were low with CT values ranging from 21 to 45 (mean: 31.78). The masses were mostly solid and uniform, while eight tumors had cystic components and four had calcification. Most of the tumors showed mild-to-moderate enhancement (Figure 1). However, untypical imaging can also be seen for some cases, such as cystic masses (Figure 2) and irregular mixed density with delayed enhancement, which can be misdiagnosed as sarcoma (Figure 3). Moreover, notably, there was one case in which the tumor was not found in plain CT.

Ultrasound is a cheap, convenient, and common way to detect abdominal and pelvic mass. GNs usually presented as hypoecho (95.45%) and some could have mixed hyperechogenic components. Half of our cases showed blood flow signals, mainly in the shape of dots or bands.

MRI was only performed in 10 patients, but in one case, it showed no obvious abnormity because of partially heavy artifact. The tumors were of hypo- to iso-intensity in T1WI, iso- to hyper-intensity in T2WI, and hyperintensity in DWI, and the ones going through enhanced MRI all reported enhancement.

On SSR imaging, no expression of the receptor was found in the tumor cells 13 out of 14 cases. However, one patient was reported to mildly express SSR in the tumor margins, which led to the clinical suspicion of pheochromocytoma before surgery even without the corresponding manifestations. In addition, the patients whose tumors were found in the adrenal area may perform MIBG adrenal medulla imaging to exclude paraganglioma, and none of them (*n* = 7) were found increased in radioactive uptake. As for PET/CT, the SUV values of the tumor were 1.4, 1.9, and 2.2, respectively, for the three patients, and only the last one indicated malignancy from the renal cortex.

In conclusion, two out of three of our cases reported the possibility of neurogenic benign tumors based on radiologic findings; 46.9% of the cases (*n* = 15) were suspected as retroperitoneal GNs as the first diagnosis, while six cases were suspected to be malignant. Others were suspected as benign tumors of other origins including lymph vessel, adrenal gland, and mesenchymal tissues.

### Laboratory Findings

Most of the patients showed relatively normal results in laboratory tests, especially for endocrine examinations and tumor markers. Nevertheless, in our cases, one young male patient had experienced elevated blood pressure 4 years before the tumor was found in the adrenal area accomplished by hypokalemia. Endocrine test showed elevated aldosterone (ALD) level and decreased plasma renin activity (PRA), and his ALD level was not suppressed by captopril. These findings supported primary hyperaldosteronism, and thus the tumor was suspected as adrenal aldosterone adenoma. However, the tumor was found not originate from adrenal tissue and was identified as GN instead by surgical pathology, and the final diagnosis was actually idiopathic hyperaldosteronism. Moreover, three more patients were found low PRA with no abnormality or symptoms, so the abnormal PRA was not enough to support other disease. However, it should also be confirmed by pathology. Another patient was found moderately high in urinate norepinephrine, and the SSR imaging showed that the receptor was mildly expressed in the tumor margins, which suggested that the tumor might be pheochromocytoma. This diagnosis was denied through biopsy and the pathology confirmed it as GN. In addition to the misdiagnosis, laboratory test could also discover complications that may be related to GN. One 19-year-old female patient had experienced menstrual disorder and body hair increase for several years, and her blood androgen, progesterone, LH, FSH, ACTH, as well as urinary free cortisol (UFC) were found increased. She was finally diagnosed as congenital adrenal hyperplasia (CAH) with non-functional GN by surgical pathology.

As for tumor markers, NSE was elevated in one patient whose CT implied irregular mix density and heterogeneously enhancement with lipid components, so there was a relatively high possibility of liposarcoma. Last, laparoscopic biopsy showed that the tumor was GN instead. The tumor was not excised because it was surrounded by vessels and hard to separate, leaving the risk of malignancy neglected by biopsy. Besides, CA724 was elevated in two patients and CA125 was found high in another patient, but they had no other evidence to support malignancy.

### Treatment

Of all the 32 patients, 2 patients underwent biopsy and chose surveillance, while the other 30 patients underwent surgery (Table 4). The two patients chose regular surveillance because the abdominal aorta and renal vessels were embedded by the mass and thus complete separation of tumor was difficult. Among the 30 surgical cases, 18 cases underwent laparoscopic operation and 12 cases performed laparotomy. In fact, the percentage of laparoscopic surgery increased from 50% (2012–2015, *n* = 6) to 66.7% (2016–2019, *n* = 12). The comparison of tumor features and surgical outcomes is summarized in Table 5; 78.95% of the tumors located in the adrenal area or near the kidney were removed through laparoscopic operation. The tumor sizes in the laparoscopic group were mostly <5 cm, which was much smaller than the laparotomic ones.

**Table 4:** Treatment of 32 cases with retroperitoneal GN.

**Strategy**	**Number (%)**
Surveillance	2 (6.25%)
Surgery	30 (93.75%)
Laparoscopy	18 (60%)
Laparotomy	12 (40%)
**Tumor location**	
Adrenal area	11 (36.67%)
Right adrenal area	7
Left darnel area	4
Renal hilum	5 (16.7%)
Other renal area	3 (10%)
Near abdominal aorta	2 (6.67%)
Near pancreas	2 (6.67%)
Near uterus	2 (6.67%)
Near liver	2 (6.67%)
**Involvement**	
Adrenal gland	4
Renal vessels	4
Inferior vena cava	2
Abdominal wall muscles	1
Iliac vessels	2
Abdominal aorta	1
Spine	1
**Resection margin**	
R0	23 (76.67%)
R1	1 (3.33%)
R2	6 (20%)

**Table 5:** Comparison between laparotomy and laparoscopic operation.

	**Laparotomy (*n* = 12)**	**Laparoscopic operation (*n* = 18)**
**Tumor location**		
Adrenal area	2 (16.67%)	9 (50%)
Renal area	2 (16.67%)	6 (33.33%)
Near abdominal aorta	1 (8.33%)	1 (5.56%)
Near pancreas	1 (8.33%)	1 (5.56%)
Near uterus	2 (16.67%)	0
Near liver	2 (16.67%)	0
Other	2	1
**Tumor size**		
Mean (max diameter)	9.2 cm	6.6 cm
<5 cm	2 (16.67%)	11 (61.11%)
5–10 cm	8 (66.67%)	5 (27.78%)
≥10 cm	2 (16.67%)	2 (11.11%)
**Surgical procedure**		
Tumor resection	10 (83.33%)	16 (88.89%)
Tumor and kidney resection	1 (8.33%)	1 (5.56%)
Tumor and gall bladder resection	1 (8.33%)	0
Adrenal resection	0	1 (5.56%)
**Complications**		
Cholecystitis	0	1

Among the 30 surgical cases, most of the tumors (63.3%) were located near the kidney, especially in the adrenal area and near renal hilum, and especially in the right adrenal area. They can also be located near aorta abdominalis (*n* = 2), pancreas (*n* = 2), uterus (*n* = 2), or liver (*n* = 2). Kidney was also resected at the same time in two cases because the renal vessels were embedded in the tumors, and gall bladder was removed in another case with complicated chronic cholecystitis. Organ involvement was seen in 13 cases, including adrenal gland, renal vessels, inferior vena cava, abdominal wall muscles, iliac vessels, abdominal aorta, and spine, while there was usually fat space between the tumors and adjacent kidney, pancreas, or uterus. R0 resection was achieved in 76.67% of the surgical cases, while R2 resection was seen in six cases and R1 was seen in one case.

### Follow-Up and Outcome

With the concept of enhanced recovery after surgery (ERAS) widely accepted, the patients who received surgery all passed flatus and fed within 5 days, mostly in 2 days (72.4%). More than half of the drainage tubes (55.17%) were removed in 3 days. The patient whose GN was around 15 cm and close to abdominal aorta and renal vessels had stayed in the ICU for 1 day after the surgery, and then fed and got rid of the drainage tube the next day. Another patient had acute cholecystitis in the 6th day after the operation and recovered in the next 2 days. The case with hand numbness and neck pain was relieved from the tumor-mass effects once the tumor was excised. No postoperative hemorrhage, limb numbness, Horner's syndrome, or intestinal obstruction was observed in our cases.

In our study, 26 patients (81.25%) was followed up. The follow-up time ranged from 6 months to 36 months, and the average time was 15.8 months. Among the 24 followed up patients with GNs excised, 18 cases had the tumors completely removed, while CT examination showed residual tumors in the other six patients. Although the operations did not achieve R0 resection, the residual tumors did not progress and two of them even shrank in subsequent examinations. No recurrence and complications were observed in all of the surgical cases. For the two cases undergoing surveillance, the tumors were evaluated by CT scanning every year. There was no discomfort, and the masses were stable during the last several years.

## Discussion

Retroperitoneal GN is a mature pNT, characterized by extremely low incidence, lack of symptom, rare recurrence, and good prognosis (11). The incidence of GN is reported around one case per million population, and retroperitoneal GN accounts for 0.72 to 1.6% of all primary retroperitoneal tumors (2). Previous studies reported different male-to-female ratios, ranging from similar morbidity (12) to 0.72–0.77 in a Japanese group (13). However, in our studied cases, the number of females is actually triple the number of males. As for predilection age, older children and young adults are found predominantly affected (14). It is suggested that GN was usually present in cases aged between 10 and 40 and that more than half of the patients were under 20 in a Japanese cohort (13, 15). The age range of our cases is 15 to 62 with a mean age of 35, which may be affected by the fact that there is no pediatric surgery department in PUMCH. So far, no other certain risk factors are found related to primary retroperitoneal GN except for possible familial predisposition (16).

Most retroperitoneal GNs are asymptomatic and nonfunctional, so they are mainly found incidentally by health examination or other disease complications (14), which agrees with our findings that nearly 66% of the cases did not have specific symptoms. However, patients can present with some nonspecific symptoms related to tumor-mass effects, for instance, abdominal pain and vomiting from compressing digestive organs, backache and scoliosis due to spinal deformity, dyspnea because of diaphragm muscle compression, as well as gait abnormality, weakness, and paresthesia owing to spinal cord compression (11, 17, 18). Among our cases, eight patients had abdominal pain and one patient had hand numbness and neck pain, which were in accordance with the tumor locations and sizes and can be explained by the mass effects. What is more, the tumors may occasionally secrete hormones including catecholamine, vasoactive intestinal peptide, and androgen, resulting in hypertension, sweating, diarrhea, or virilization (13, 19–23). These suggest that GNs can contain functional cells or mix with adenoma tissues, but the exact mechanism remains to be explored. In our study, the 19-year-old female patient presented with the features of CAH once suspected as functional GN that led to adrenal hyperplasia, but finally, the surgical pathology found no functional cells in the GN.

As retroperitoneal GNs lack specific symptoms and laboratory findings, clinical diagnosis mainly depends on radiological examination. Typically, the mass is well-circumscribed, oval, or oval lobulated with diameters ranging from 3 to 10 cm and tends to surround major blood vessels without compression or occlusion (19, 24). Masses larger than 10 cm were only seen in five cases in our study. Among the imaging methods, ultrasound and CT are the most used for retroperitoneal GNs. Ultrasound normally reveals a homogeneous or slightly heterogeneous, hypoechoic, solid, and hypovascular mass (25), but our studies suggested 50% of the masses exhibited varied blood flow signals. On plain CT, previous studies revealed that the densities varied from 15 to 38 HU with a mean of around 30 HU, and fine and punctate calcifications can be seen in some cases instead of amorphous and coarse ones seen in neuroblastomas (24–26). Late-phase enhancement in a range of 10–20 HU has been reported for GNs (27). The radiologic findings in most of our cases fulfill these characteristics, but in one case, the tumor was not discovered by plain CT. When it comes to the diagnosis based on CT findings by radiologists, two out of three of our cases reported the possibility of neurogenic benign tumors, while six cases were suspected to be malignant. Other tumors that are relatively hard to be distinguished from GN by CT include adrenal tumors, lymphatic lesions, leiomyosarcoma, liposarcoma, and tumors originating from adnexa and embryo. The radiological misdiagnosis is mainly due to the atypical performance of the mass and lack of experience of the clinical radiologists. For MR imaging, GN tends to be shown as low intensity on T1WI and heterogeneous high intensity on T2WI with similar enhancement features as in CT (14). There are also other imaging methods that can be used to exclude functional tumors or malignancy. For example, SSR imaging is a common method for screening neuroendocrine tumors with good sensibility and specificity (28). MIBG imaging is used to identify neuroblastoma, paraganglioma, and pheochromocytoma (29). Among our cases, PET/CT was the least used for GN patients, which helped to locate malignant lesions (29). The methods usually show negative findings for most GN masses and have been rarely studied in GN cases. As is shown in our patients, only 2 cases had clinically significant abnormal findings in these imaging methods, which required pathological confirmation for final diagnosis.

Previous studies revealed that most of the cases with retroperitoneal masses were actually malignant and 90% of the malignancies were sarcoma, while the common benign tumors included neurogenic tumors, paragangliomas, fibromas, angiomyolipomas, and lipomas (9, 30). We found that there were no special and featured findings in laboratory examination for the GN patients. For differential diagnosis, the patients usually received endocrine tests and checked tumor markers, but no abnormal results were seen in most of the patients. However, since GN lacks specific clinical manifestations and laboratory tests, the ones with abnormal laboratory or unusual radiological findings would be easily misdiagnosed as other tumors including kidney, ovarian, and adrenal masses (9, 31–33). Other neurogenic tumors such as neuroblastomas and ganglioneuroblastomas should also be considered, especially for children (34). Besides, the composite ones mixed with other tumor cells such as pheochromocytoma or lipoma were rarely reported, which could be confusing and diagnosed as other tumors (23, 35). Among our cases, adrenal aldosterone adenoma, pheochromocytoma, and liposarcoma were, respectively, suspected in three patients in preoperative diagnosis based on the symptoms and examination results. The possibility of malignant or functional tumors could not be ruled out in others with untypical laboratory or radiological findings. Therefore, histopathology is still the gold standard for GN diagnosis (36). In conclusion, preoperative differential diagnosis is difficult for retroperitoneal GN due to radiological confusion with other tumors as well as untypical symptoms and laboratory tests. It should always be kept in mind that a retroperitoneal mass may be a GN in clinical practice, and the definite diagnosis can only be made through pathological evaluation after surgery or biopsy.

Surgery is an effective way for retroperitoneal GN treatment, and complete resection is widely accepted as an adequate therapy with no recurrence and good prognosis (37). Besides, our cases imply that with the development of minimally invasive surgery, more surgeons chose to remove the small GNs that are near adrenal gland or kidney through laparoscopic operation or even robotic surgery. The overall survival rate can reach 90–100% in previous reports, but relatively severe complications from surgery are not unusual due to proximity to vessels, spine, and other organs, which include hemorrhage, Horner's syndrome, intestinal obstruction, and even death (3). A retrospective series study of 146 children with GN reveals that around 15% of the cases (*n* = 22) suffered from surgery-related complications, and among them, two were fatal and seven were severe (38). The residual tumors remained stable without progression and malignant transformation (38). Besides, another study of 24 children with chest, abdomen, and pelvic GNs found that seven of the patients had complications after surgery, while no tumor progression and recurrence was observed in the cases with incomplete resection or surveillance at 33.5 ± 40 months follow-up (39). Similar findings were seen in a Spanish research in that among 24 GN cases, 25% of the patients went through postoperative complications and the residual ones did not regrow or become malignant after a follow-up of 84 (1–194) months (40). In a word, these previous studies suggest that although R0 resection can be hard and even impossible for some cases, tumor progression and recurrence have not been seen with residual lesions or surveillance, leaving the debate whether surgical excision is necessary for GN patients. Therefore, less radical surgery or even surveillance after secured diagnosis was recommended by De Bernadi and Retrosi for childhood GNs to reduce surgery-related morbidity and death to improve the overall outcomes. However, it should be noted that these findings were not aimed for retroperitoneal GN and the cases were all children. There is still not enough evidence to guide the surgery options for adult retroperitoneal GN. In fact, although biopsy is a choice for pathological diagnosis, it can lead to inconclusive results due to limited biopsy tissue and frequent association with other components (24, 41). A large series study concerning presacral GNs suggests that fine-needle aspiration (FNA) biopsy failed in diagnosis in several patients (41). What is more, studies suggest the rare capacity of GN to develop into NB and the small possibility of malignant transformation, which indicates the possible risk of incomplete resection and necessity for radical surgery for some patients (42–45).

In our retroperitoneal GN cases, 2 out of 32 patients chose surveillance due to vessel embeddedness. However, CT imaging of the two patients showed irregular mass with rough borders and lipid components, which implied the possibility of malignancy. Therefore, considering the limitation of biopsy site and size, the tumors still had the risk of cancerization in spite of the benign pathology, which had been informed to the patients. Since there were no discomfort and renal dysfunction, patients refused surgery and received surveillance. They were followed up, and it turns out that the tumors have been stable for years. Among the other 24 postoperative follow-up patients (mean = 15.8 months), six cases were incompletely resected due to either artery or spine involvement or the large size of the fused masses. No tumor progression and malignant transformation was seen in spite of incomplete excision in the six cases, and two of them even shrank in the following examinations. Kidney was removed in two cases for complete resection of the tumor, and gall bladder was resected in a patient with chronic cholecystitis and tumor was located near the gall bladder. Major vascular resection with prosthetic replacement was also seen for a recurrent retroperitoneal GN (46), but it was recommended to be considered as the last resort and used for recurrent tumors (47). As metastasis and recurrence are rare, previous studies suggest to avoid unnecessary wide excisions and that preservation of surrounding organs and vascular structures should be achieved as far as possible (47, 48). Given the fact that recurrence is rarely observed after palliative operation and that combined organ or vascular resection may result in complications, incomplete resection and preservation of neighboring structures should be considered for the stable GNs involving major vessels or organs, unless the tumor is recurrent or potentially invasive. Thanks to the prudent surgical manipulation in our hospital, no severe complications were reported, including the ones with combined kidney, adrenal gland, or gall bladder resection. Nevertheless, limited by the follow-up period and sample capacity, this observation was not enough to confirm the outcomes of different therapy strategies for retroperitoneal GNs. The rationale for GN excision remains to be further explored.

In conclusion, although complete resection is an adequate therapy, the surgery may result in severe complications when the mass is adjacent to major vessels, nerves, or important organs. Therefore, incomplete resection is suitable for the tumors hard to separate, and surgical pathology can be then used for definite diagnosis, which is more convincing than percutaneous biopsy. Besides, preoperative percutaneous biopsy can help to diagnose retroperitoneal GN with relatively high sensitivity, especially when radiological imaging is untypical. However, it should be noted that there is still a low possibility of false negatives and even long-term malignant transformation. Surveillance can be considered for the cases with high risks of postoperative complication who are diagnosed as GN by biopsy, especially for the patients without distinct symptoms. All the patients should receive regular re-examinations and follow-up to avoid tumor progression or recurrent.

As for other therapy options, due to the benign natures of GN, adjuvant systemic chemotherapy or local radiotherapy are not considered in clinical practice (34). Actually, radiotherapy is a common cause inducing the secondary GN (49, 50). So far, surgical resection is still the only curative therapy without the need for chemotherapy or radiotherapy (51, 52).

## Conclusions

Retroperitoneal GN is a rare benign tumor with excellent prognosis, mainly seen in children and young adults. Most of the tumors are asymptomatic without specific laboratory findings, while nonspecific tumor-mass effects and functional mass can be seen in some cases. The clinical preoperative diagnosis mainly depends on radiological imaging, which shows low density <10 cm with a clear border and an embedded growth pattern. Differential diagnosis can be hard for the ones with untypical performance and features, and pathology is the gold standard for GN diagnosis. Surgery is widely accepted as an effective therapy with possible complications, and rare tumor progression is seen in less radical surgery or surveillance. More investigations are needed to further explore the rationale and outcomes of resection for retroperitoneal GN.

## Data Availability Statement

The raw data supporting the conclusions of this article will be made available by the authors, without undue reservation.

## Ethics Statement

Written informed consent was obtained from the individuals, and minors' legal guardian/next of kin, for the publication of any potentially identifiable images or data included in this article.

## Author Contributions

JX and ZZ designed the study. ZZ collected and analyzed the data and wrote the paper. JX revised the paper. All authors contributed to the article and approved the submitted version.

## Conflict of Interest

The authors declare that the research was conducted in the absence of any commercial or financial relationships that could be construed as a potential conflict of interest.

## Nomenclature

GN: Ganglioneuroma

pNTs: peripheral neuroblastic tumors

NB: neuroblastoma

GNB-N: ganglioneuroblastoma-nodular

GNB-I: ganglioneuroblastoma-intermixed

INPC: International Neuroblastoma Pathology Classification

PUMCH: Peking Union Medical College Hospital

CTD: connective tissue disease

CT: computed tomography

MRI: magnetic resonance imaging

SSR: somatostatin receptor

ALD: aldosterone

PRA: plasma renin activity

UFC: urinary free cortisol

CAH: congenital adrenal hyperplasia

ERAS: enhanced recovery after surgery

FNA: fine-needle aspiration
